# Radiating pain: venom has contributed to the diversification of the largest radiations of vertebrate and invertebrate animals

**DOI:** 10.1186/s12862-021-01880-z

**Published:** 2021-08-03

**Authors:** Kevin Arbuckle, Richard J. Harris

**Affiliations:** 1 Department of Biosciences, College of Science, Swansea University, Swansea, SA2 8PP UK; 2 Venom Evolution Lab, School of Biological Sciences, University of Queensland, Saint Lucia, QLD 4072 Australia

**Keywords:** Diversification rates, Venom, Insects, Fishes, Biodiversity, Species richness

## Abstract

**Background:**

Understanding drivers of animal biodiversity has been a longstanding aim in evolutionary biology. Insects and fishes represent the largest lineages of invertebrates and vertebrates respectively, and consequently many ideas have been proposed to explain this diversity. Natural enemy interactions are often important in diversification dynamics, and key traits that mediate such interactions may therefore have an important role in explaining organismal diversity. Venom is one such trait which is intricately bound in antagonistic coevolution and has recently been shown to be associated with increased diversification rates in tetrapods. Despite ~ 10% of fish families and ~ 16% of insect families containing venomous species, the role that venom may play in these two superradiations remains unknown.

**Results:**

In this paper we take a broad family-level phylogenetic perspective and show that variation in diversification rates are the main cause of variations in species richness in both insects and fishes, and that venomous families have diversification rates twice as high as non-venomous families. Furthermore, we estimate that venom was present in ~ 10% and ~ 14% of the evolutionary history of fishes and insects respectively.

**Conclusions:**

Consequently, we provide evidence that venom has played a role in generating the remarkable diversity in the largest vertebrate and invertebrate radiations.

**Supplementary Information:**

The online version contains supplementary material available at 10.1186/s12862-021-01880-z.

## Background

Biodiversity is unevenly spread across the tree of life, with some groups being far more diverse than others [1, 2]. Understanding why some taxa have particularly high species richness is an important step in revealing the underlying patterns and processes governing the origin and maintenance of biodiversity. At a broad level, the accumulation of species richness in a lineage is a result of two factors: (net) diversification rate (speciation rate minus extinction rate) and age [3]. Both of these are inevitably going to play a role because, for a given diversification rate, older taxa have had more time to accumulate species, whereas for a given age a clade with a higher diversification rate will be producing more species in that timeframe. Nevertheless, the relative influence of diversification rates and age on observed patterns of variation in species richness appears to vary between clades and perhaps with phylogenetic scale (e.g. [3–6]).

Although clade age is an independent factor in species richness, diversification rates can in principle be influenced by a range of organismal traits. Such traits can influence diversification rates in a multitude of ways, but two important forms are commonly discussed: dead end traits [7, 80, 81], which evolve due to short term gains but lead to increased extinction rates, and key innovations [7, 82, 83]. The term ‘key innovation’ has been inconsistently defined, leading to some confusion, but most commonly refers to a trait which increases the diversification rate of the lineages which possess it [8]. Key innovations are typically expected to operate by increasing ecological opportunity, allowing further exploration of niche space and inducing a greater probability of speciation, or in some cases act as direct triggers of speciation [7].

Traits which are involved in natural enemy interactions are prime candidates for key innovations [9, 10]. This is due in part to Ehrlich and Raven’s [11] idea that natural enemies act as strong ecological constraints on diversification. The ability of each party to ‘break’ those constraints (e.g. by a novel defence mechanism or an adaptation to dealing with formidable prey that is difficult to subdue) should therefore be associated with niche expansion into an adaptive zone of ‘enemy-free space’. This concept has subsequently been called ‘escape-and-radiate’ coevolution [12] and is the primary explanation for why antagonistic coevolutionary arms races may promote speciation, and consequently diversification.

Animal venoms are emerging as a potential key adaptation across a wide range of taxa. Under the idea of escape-and-radiate dynamics this is perhaps not surprising since antagonistic coevolution is a unifying feature of all venoms [13]. Moreover, previous empirical studies have found that venom is associated with increased diversification in tetrapods [14] and that a venomous genus of blenny (fang blennies, *Meiacanthus*) experienced a rapid upshift in diversification rate [15]. Nevertheless, Blanchard and Moreau [16] found that venomous stings in ants were associated with reduced diversification rates, likely as a result of trade-offs with other defensive traits which increased diversification. Furthermore, although dealing with poison rather than venom, we previously demonstrated decreased diversification rates despite increased speciation rates (via increased extinction rates) in poisonous amphibians [17]. Hence, it is important to evaluate the generality of the predicted relationship of increased diversification in venomous animals, since escape-and-radiate dynamics leading to increased speciation rates may not be realised in net diversification rates due to trade-offs or altered extinction rates.

Insects and fishes represent the most species-rich lineages of invertebrates and vertebrates respectively, containing ~ 1 million (~ 75%) described species of invertebrates and 31,269 (48%) described species of vertebrates [2]. We note that we have considered ‘fishes’ to be a distinct lineage for the purposes of this paper despite tetrapods being nested within this group. However, although fishes are not a clade, but rather a grade of non-tetrapod vertebrates, they are highly ecologically distinct from tetrapods and as such it is common for macroevolutionary studies to consider such groups separately from their nested (but highly ecologically divergent) clades [e.g. 73–75]. Moreover, some of the questions we test here have already been examined in tetrapods and we wish to understand how general the answers are beyond this group. Notably, compared to the tetrapods and other groups which have previously been studied in the context of venom-associated diversification rates (as discussed above), the large phylogenetic scale of both insects and fishes has the potential to uncover older origins of venom. This could be important because the molecular evolution of the toxins within animal venoms has been shown to follow a ‘two-speed’ model [76]. In this model, relatively rapid adaptive evolution of venom toxins occurs soon after the origin of venom which diversifies the venom composition, followed by slower rates of toxin evolution which maintains the molecular composition of venom. Hence, it is possible that arms races and related coevolutionary explanations for venom-associated diversification become less effective at promoting diversification in older lineages where rates of molecular evolution are slower.

Due to the remarkable species diversity of fishes and insects, researchers have proposed several factors that may have increased diversification rates in these groups (but note that since fishes have a similar species richness to tetrapods, yet are older, they may not necessarily have increased diversification rates compared to tetrapods). For insects, most explanations have revolved around the role of herbivory [18], metamorphosis [19, 20], wings/flight [19], and the general insect body plan [19]. Of those the clearest support has been found for a role of herbivory and metamorphosis, with a more complex role for wings that involve effects on both speciation and extinction rates [19]. In contrast, proposed factors increasing diversification rates of fishes have been related to habitat, finding faster diversification rates in freshwater fish [21] (but see [22]), reef fish [21], and pelagic (cf. benthic) freshwater habitats [23].

Notwithstanding the proposed influences on diversification rate above, the potential role of venom in contributing to the great diversity of fishes and insects has not yet been investigated. This is despite many fishes and insects being venomous and in both groups venom is thought to have arisen multiple times independently [13, 24–26]. Fish venoms have evolved, with few exceptions, in a defensive role and their venoms typically cause extreme pain resulting from both direct and indirect (via cytolytic effects) toxic actions [26, 27]. Insect venoms are also commonly defensive, including the painful envenomations from clinically important groups such as social hymenopterans and lepidopterans [28, 29], but in many groups function primarily in feeding [13, 25]. As with all venoms, fish and insect venoms are tightly linked to coevolutionary interactions such as predator–prey relationships. Following the discussion above, this may lead to impacts on diversification rates that could contribute towards explaining the high species richness of fishes and insects. Note that we neither claim nor expect venom to provide the predominant explanation for the diversity of these large groups. Diversification dynamics are the result of many influences and we would only rarely (if ever) expect a single factor to explain most of the variation in species richness of any large clade. Nevertheless, as a role for venom in generating the diversity of insects and fishes has not been tested, despite reasons to believe it could have an influence, we aim to evaluate whether it may have contributed to the observed species-richness.

In this paper, we take a broad, family-level, phylogenetic perspective to test whether venom has contributed to the biodiversity of insects and fishes via increasing diversification rate. Specifically, we first test whether diversification rate (rather than clade age) is the main determinant of species richness in these groups. Second, we test whether the presence of venom is associated with higher diversification rates. Third, we evaluate how important any relationships may have been across the evolutionary history of the lineages by testing how many times venom has evolved in each group and how much of their evolutionary history venom has been present for.

## Methods

### Data collection

We obtained family-level time-calibrated phylogenies of insects and fishes from the TimeTree database [30]. Because fishes are not a monophyletic clade we first downloaded a family-level phylogeny of Gnathostomata and then pruned tetrapod families from the tree in the R package ‘ape’ [31], resulting in a phylogeny of chondrichthyan and (non-tetrapod) osteichthyan fish families. These two phylogenies were used for all comparative analyses herein. The phylogenies obtained from the TimeTree database provided wide coverage of families in both groups, but have the limitation that they include some polytomies. We follow recent studies (e.g. [77]) in addressing this by adding a small value to branch lengths (0.5my) and then adjusting the branch lengths to ensure the tree remains ultrametric using the force.ultrametric function in the R package ‘phytools’ [57]. The impact of polytomies in most comparative methods is still poorly understood, but arbitrarily resolving them tends to lead to inflation of rates of phenotypic evolution [78]. Nevertheless, such issues are likely of limited concern for diversification analyses [78] and other studies have found that small degrees of tree misspecification, including polytomies, has limited impact on phylogenetic regression approaches [79]. Hence, although we acknowledge the inherent limitations of polytomies herein, we believe that our study should be reasonably robust to this problem, especially with the combination of methods employed that should be affected in different ways.

For each insect and fish family we collected data on total species diversity from existing literature and biodiversity databases [21, 24, 32–36]. We also recorded whether each family contained venomous species (as a binary presence/absence variable) based on existing literature [24, 37–44]. The literature reviewed is typically consistent in its consideration of which organisms are venomous, and typically follows accepted definitions, e.g. possessing “a secretion, produced in a specialised gland in a target animal through the infliction of a wound…which contains molecules that disrupt normal physiological or biochemical processes so as to facilitate feeding or defense by the producing animal” [45].

We note that our family-level data coding of venom intrinsically assumes that all species of a family are either venomous or non-venomous (but not a mixture of both). We acknowledge that this is a limitation, as in previous studies of fish venom such as [24] which stated that “if the distribution of venom within a small clade that lacks diagnosed subgroups (e.g., surgeonfishes in the genus *Acanthurus*) was unclear because both venomous and non-venomous forms have been noted, a range is given (Supplementary Information)”. We examine the robustness to this potential issue by re-running all our models on a reduced dataset excluding families with intrafamily variation in venom presence. We report the results of those analyses in Additional file 1: Table S3, and note that they were consistent with the analyses we present herein, perhaps unsurprising given that 96.1% (441/459) of fish families and 99.7% (883/886) of insect families in our data show no uncertainty in the sense that all members of a given family are considered either venomous or not. Hence, we acknowledge that this can be an issue in principle, but believe our results are robust to the minor deviations from our approach that appear to be present in nature and reflects the standard classifications of venomous groups in the literature. In essence, venom seems to be more-or-less a family-level trait in the taxonomic groups studied here (with a small proportion of exceptions) and so our coding approach is reasonable.

Finally, we extracted the stem ages of each family from the TimeTree database to allow us to estimate diversification rates for each family using the method-of-moments estimator [46] implemented in ‘geiger’ v2.0.6.2 [47]. We provided the estimator function with the total species richness of each family as the diversity of the clade. We preferred stem ages to crown ages for two reasons: (1) stem ages were available for all families but crown ages cannot be estimated for monotypic families and are more susceptible to inaccuracies when basal lineages within the family are poorly sampled; and (2) perhaps in part for reasons related to point 1, simulation studies have found that diversification rates estimated from stem ages are generally more accurate than those estimated from crown ages [48]. The method-of-moments estimator requires specification of relative extinction rates (ε), which are typically unknown. Consequently, following previous guidelines and usage (e.g. [46, 48]), we estimated diversification rates assuming no extinction (ε = 0), medium (ε = 0.5), and extremely high (ε = 0.9) relative extinction rates. Where we refer to ‘diversification rates’ in descriptions of analyses we ran three sets (one for each of our relative extinction rates), but in almost all cases the results were qualitatively the same regardless of which version we used, so we report only results based on ε = 0.5 in the main paper and highlight when any results differed from this. Model outputs from analyses using assumptions of no and extremely high levels of extinction are included in Additional file 1: Tables S1 and S2.

### Analyses

To test whether clade age or diversification rate is more important in generating the extant species richness of insects and fishes we used phylogenetic linear regression as implemented in the R package ‘phylolm’ v2.6 [49]. We modelled log(species richness) as a function of diversification rate, age (as a quadratic term to account for non-linear effects of age on species richness [50]), and venom. We included venom in these models to test whether venom was more likely to evolve in bigger clades after accounting for diversification rate and age. This would indicate that venom may simply be associated with species richness due to more species giving more opportunities for venom to arise. For these models, and all other phylogenetic linear regressions in this paper, we first tested which of the trait evolution models available in ‘phylolm’ (plus a non-phylogenetic general linear model) provided the best regression fit based on Akaike’s information criterion (AIC), and used this model for further inference. The available trait evolution models tested were Brownian motion, Ornstein–Uhlenbeck (OU) with a random or fixed root (we arbitrarily preferred random roots when fit models were identical), lambda, kappa, delta, early burst, or trend. For models of species richness, OU with a random root best fit the insect dataset and lambda best fit the fishes dataset.

To test whether the presence of venom predicts diversification rates (controlling for clade age) we again used phylogenetic linear regressions in ‘phylolm’. For these we modelled diversification rate as a function of venom and age (as a quadratic term to account for the expected non-linear effects of age [50]), based on the best fitting trait evolution models of OU with a random root for fishes and kappa for insects. As an independent test for a relationship between diversification rate and venom we also used a sister group approach, specifically the richness Yule test developed by Paradis [51] as a more powerful alternative to other sister group methods. We implemented the sister group analyses in the R package ‘ape’ v5.3 [31] and used the total species richness in each pair of sister clades, not simply number of descendent tips in the phylogeny (which only represents number of taxonomic families, not species richness). The richness Yule test considers differences in species richness between two sister clades which differ in the binary trait of interest (in this case the presence of venom), as with all sister group methods, and compares the difference in the two sister lineages to the expectation under a Yule model.

As one more test for an association between venom and diversification rates we also fit a series of BiSSE [52] models to the two datasets using diversitree v0.9.11 [53] and broadly following the approach in [17]. BiSSE attempts to test for different speciation (λ) and/or extinction (μ) rates in relation to a binary trait (in our case the presence of venom) while accounting for unequal transition rates (*q*). Briefly, to each of the fish and insect datasets we fit five BiSSE models: an unconstrained model, a fully constrained model (equal diversification and transition rates), a diversification constrained model (equal diversification rates but transition rates are free to differ), a speciation constrained model (equal speciation rates but extinction and transition rates can vary), and an extinction constrained model (equal extinction rates but speciation and transition rates can vary). To account for the tendency of BiSSE models to favour trait-dependent models due to diversification rate heterogeneity not associated with the trait [84], we also fit a hidden rates (HiSSE) model in hisse v1.9.1 [85] similar to the full BiSSE model but including a hidden state to allow for rate variation. The best model was chosen based on the lowest AIC and parameters of this model were inspected to enable inference. We then simulated 1000 trees plus traits based on our best model for each tree and evaluated the fit of this model by comparing 1) the proportion of venomous species and 2) the number of tips in each state from our simulations to the observed values. We note that we treat the results of our BiSSE analyses only as confirmatory analyses in combination with our other sets, partly because such an analysis is essentially treating the families as the tips and hence families with vastly different species richness are treated equally in this analysis. Consequently, this method is addressing a slightly different question by asking whether venom may be associated with diversification at higher phylogenetic scales (family diversity rather than species diversity). However, using a method with another different set of assumptions and looking at a different scale may help support other analyses by confirming the generality of the results. We note that methods for trait-based diversification analyses are currently a highly debated issue in comparative biology with different authors favouring different approaches. However, we suggest that our three independent methods, which have very different assumptions and limitations, give concordant results (see below) and hence collectively provide a body of evidence supporting our conclusions. We further highlight that despite some previous claims, the method-of-moments estimator for diversification rates as used herein does not require constant rates of diversification within clades [48], and we control for time-dependent rate variation between clades by including clade age in our models.

Finally, we used ancestral state estimation to infer the number of origins of venom in insects and fishes and how much of their evolutionary history venom was present. We note that because we used family-level phylogenies, the number of origins estimated here represents a minimum, since it is possible that in some cases venom has evolved multiple times within a given family. We used stochastic mapping [54–56], implemented in the R package ‘phytools’ [57], to estimate ancestral states based on 1000 simulations and a general (ARD; all rates different) model of trait evolution to allow rates of gain and loss of venom to differ. As we were interested in the number of origins of venom rather than the states at each node, and also due to practical difficulties of plotting estimates at all nodes on large phylogenies, we plot our results as the inferred gains on branches where this transition was estimated to occur.

## Results and discussion

We found that 46 of 459 (10%) fish families and 145 of 886 (16%) insect families were venomous. The median species richness for venomous insect families was 277 species compared to 107 for non-venomous families. For fishes, venomous families had a median of 40 species compared to 18 species in non-venomous families. Similarly, median net diversification rate for venomous insect families was 0.040 compared to 0.029 for non-venomous families, and for venomous fish families median net diversification rate was 0.041 compared to 0.033 for non-venomous families (all calculated from method-of-moments estimators).

Unsurprisingly, both diversification rate and age significantly predicted species richness for insect and fish families, but importantly we found a substantially larger effect of diversification rate (Table 1). This demonstrates that the variation in diversification rates (rather than clade age) is the main driver of variation in species richness of fishes and insects. Previous studies have varied in support for diversification rates vs clade age as a main predictor of species richness [3–5], though these studies vary in taxonomic breadth and the particular clades analysed which is likely an important moderating factor. Notably, a review of predictors of species richness which included consideration of various groups of insect families, and hence being among the best comparisons for our study, found results consistent with our findings [6].

**Table 1:** Model output predicting species richness showing that all included explanatory variables were significant predictors of diversity, though diversification rate dominated

	Coefficient	SE	t	P
*Fishes*				
Intercept	− 1.0392	0.7996	− 1.2996	0.1944
Venom	0.5201	0.2242	2.3202	0.0208
Diversification rate	29.6560	1.3786	21.5108	< 2.2 × 10^–16^
Clade age	0.0436	0.0048	9.1523	< 2.2 × 10^–16^
Clade age (quadratic)	− 0.0001	0.00002	− 6.9864	1.0 × 10^–11^
*Insects*				
Intercept	− 1.1889	0.3980	− 2.9869	0.0029
Venom	0.5777	0.1694	3.4094	0.0007
Diversification rate	39.5480	1.7124	23.0944	< 2.2 × 10^–16^
Clade age	0.0515	0.0046	11.1436	< 2.2 × 10^–16^
Clade age (quadratic)	− 0.0001	0.00001	− 9.0802	< 2.2 × 10^–16^

Lambda parameter for fish model was estimated to be 0.784, the estimated parameters from the OU model for insects were α = 0.016 (phylogenetic half-life of 43.7my) and σ^2^ = 0.098. Results shown for diversification rate based on ε = 0.5, see Additional file 1: Table S1 for equivalent model outputs for ε = 0 and ε = 0.9

We also found that the ages of venomous and non-venomous families were similar for both fishes and insects (Additional file 1: Fig. S1), suggesting that clade age is not a potential mediating factor of any relationship between venom and diversity. Venomous families tended to be associated with more species rich families even after accounting for diversification rate and age (Table 1). However, this independent effect of venom is small (Additional file 1: Fig. S2) and so more opportunity for venom to evolve in larger families cannot readily account for any effects of venom on diversification rates.

**Fig. 1:**
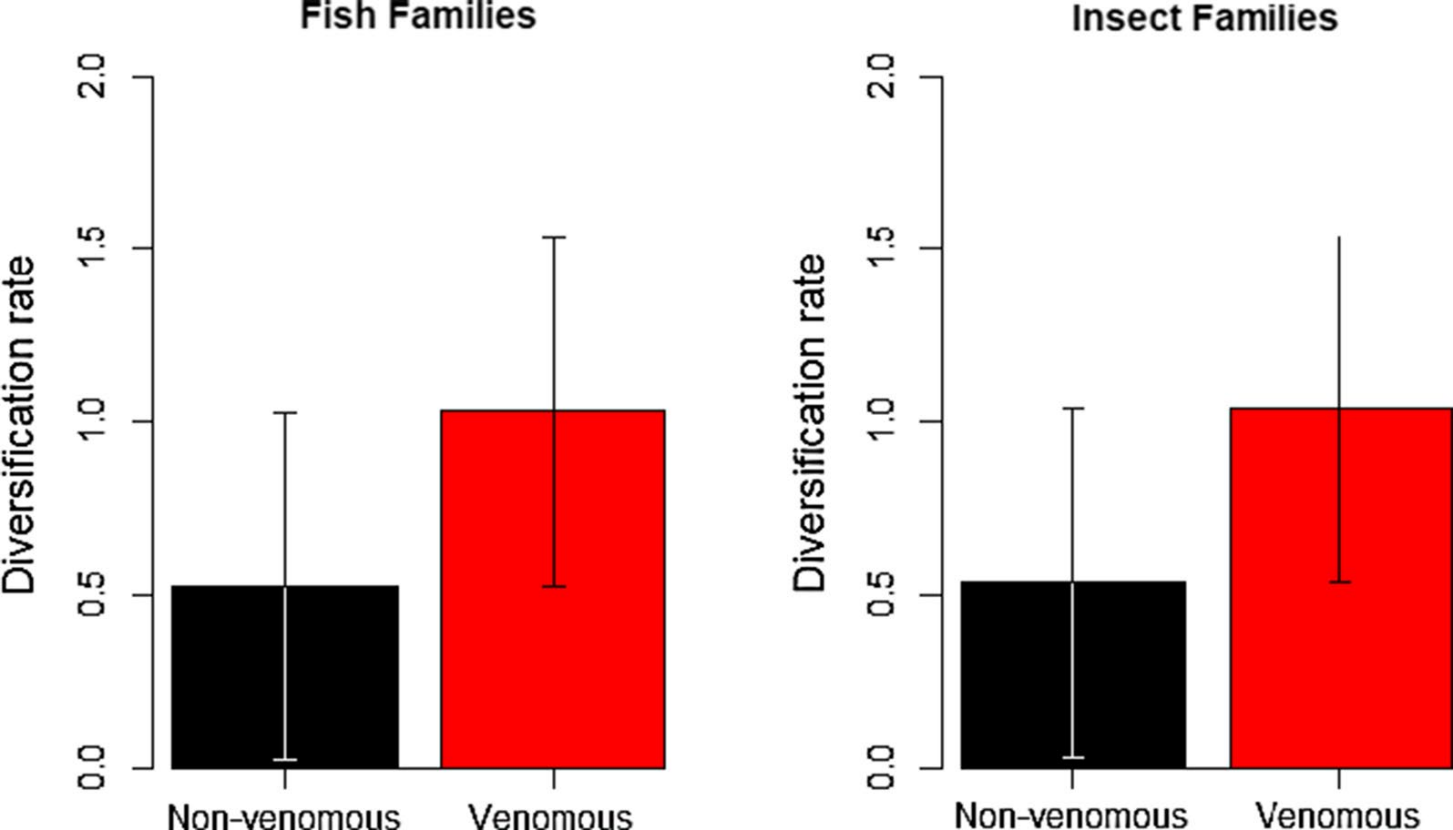
Venomous families have a net diversification rate approximately double that of non-venomous families for both fishes and insects. Diversification rates and standard errors are based on estimated parameters from a model accounting for clade age which found that the differences shown here are statistically significant despite the relatively wide confidence intervals (Table 2)

Our phylogenetic linear regression analyses provided evidence that venomous families have higher diversification rates than non-venomous families in both fishes and insects (Table 2, Fig. 1). We note that the only exception to this result is for insects assuming high relative extinction rates (ε = 0.9), in which case venom is a non-significant predictor of diversification rates (Additional file 1: Table S2). This is perhaps reason for caution as this is our only result that is sensitive to relative extinction rate, but previous estimates of relative extinction rates for insects have been very low and near zero [33], far from reaching 0.9. Our sister group analyses further support our findings of an increased diversification rate in venomous families of both fishes (*χ*^2^ = 15.91, df = 1, P = 6.6 × 10^–5^) and insects (*χ*^2^ = 12.65, df = 1, P = 0.0004). Although not controlling for age of sister clades, venomous fish families had a mean species richness ~ 5.3 times that of their non-venomous sister groups while venomous insect families were a mean of ~ 4.0 times more species rich than their non-venomous sister group. Our BiSSE analyses found strong support for the extinction constrained model being the best in our sets for both fishes and insects (ΔAIC between this and the next best model in fishes is 37.10 and in insects is 426.15, except the HiSSE model for insects, which is very similar at ΔAIC = 0.42, but as a more complex model we prefer the simpler constrained BiSSE model). This suggests that venom is associated with speciation, but not extinction rates, and the magnitudes of the effects are similar in both groups (Table 3). Our parameter estimates suggest that venomous lineages have a speciation rate two orders of magnitude larger than non-venomous lineages when accounting for transitions rates (which are estimated as an order of magnitude higher for loss than gain of venom). However, note that model adequacy tests aiming to assess the absolute fit of these models to the data found that the family-level diversity of insects was greatly underestimated by these models, with fish diversity being slightly underestimated and broadly appropriate proportions of venomous species were estimated (Additional file 1: Figure S5). While our general comparison may still hold (showing that venomous lineages diversify more rapidly), parameter values from those models should be interpreted with great caution.

**Table 2:** Model output predicting diversification rate showing that venom is associated with faster diversification

	Coefficient	SE	t	P
*Fishes*				
Intercept	0.0973	0.0057	16.9783	< 2.2 × 10^–16^
Venom	0.0135	0.0058	2.3092	0.0214
Clade age	− 0.0010	0.0001	− 9.6325	< 2.2 × 10^–16^
Clade age (quadratic)	2.4 × 10^–6^	3.9 × 10^–7^	6.1143	2.1 × 10^–9^
*Insects*				
Intercept	0.1357	0.0216	6.2873	5.1 × 10^–10^
Venom	0.0207	0.0058	3.5674	0.0004
Clade age	− 0.0012	0.0001	− 10.7577	< 2.2 × 10^–16^
Clade age (quadratic)	2.7 × 10^–6^	3.2 × 10^–7^	8.3214	3.3 × 10^–16^

The estimated parameters from the OU model for fishes were α = 0.050 (phylogenetic half-life of 13.8 my) and σ^2^ = 0.0001, whereas kappa was estimated to be 3.5 × 10^–8^ in the insect model. Results shown for diversification rate based on ε = 0.5, see Additional file 1: Table S2 for equivalent model outputs for ε = 0 and ε = 0.9

**Table 3:** Parameter estimates for the best BiSSE model in our model set for each taxonomic group

Taxa	λ_venom_	λ_novenom_	μ	*q*_gain_	*q*_loss_
Fishes	0.052	9.81 × 10^–4^	1.93 × 10^–7^	0.007	0.076
Insects	0.022	2.15 × 10^–4^	1.45 × 10^–6^	0.004	0.030

For both fishes and insects the best model contain unequal speciation rates (higher for venomous lineages), equal extinction rates, and unequal transition rates (higher rates of loss than gain of venom). λ_venom_ = speciation rate of venomous lineages, λ_novenom_ = speciation rate of non-venomous lineages, μ = extinction rate, *q*_gain_ = rate of gain of venom, *q*_loss_ = rate of loss of venom

Our results supporting higher diversification rates in venomous fishes and insects are consistent with similar findings in tetrapods [14] and in one study of fishes that focussed on a single genus (*Meiacanthus* [15]). However, such a relationship has not been found in all cases; for instance Blanchard and Moreau [16] found no evidence for venomous stingers being associated with faster diversification in a family of insects (Formicidae). Despite suggestions in the literature that venom may be important in the diversification patterns of fishes overall [26], our study is the first to investigate the question across the whole lineage (and that of insects). Since work on individual genera or families have yielded conflicting results, the broad-scale approach we use here represents a key advance in determining general patterns concerning the diversification of these large lineages. The link between chemical weaponry and diversification has typically revolved around chemical defences (e.g. [17]). In the case of defensive venoms we expect escape-and-radiate dynamics to provide the mechanism [9, 11], and this would therefore apply to fishes in general and some major groups of insects (e.g. social hymenopterans). However, many venomous insects primarily use their venom for prey capture/subjugation [13]. In these groups we suggest that venom may have allowed a broader range of prey to be consumed, such as bigger or more dangerous prey [58, 59], and this niche expansion may have led to increased diversification rates, perhaps by providing additional opportunities for specialism on an increased variety of prey options [60, 61].

Interestingly, not only was the best fitting trait evolution model for our phylogenetic linear regressions of insect diversification rates found to be kappa, but the estimated parameter value was extremely low, in fact almost zero (Tables 2 and Additional file 1: Table S2). This is consistent with diversification rates of insects evolving under a punctuated equilibrium scenario [62], wherein rates can change in bursts at the origination of new lineages but not continuously along branches of the phylogenetic tree. To our knowledge this has not been previously reported in the literature. Such a pattern of punctuated evolution of diversification rates may pose substantial problems for robustly testing for key innovations. Many suggested key innovations of insects are unique traits in the sense of having a single origin at the base of a large clade and have been retained almost throughout the descendent lineage [19]. If diversification rate shifts coincide with origins of new lineages then every new lineage has an equal chance of an upshift or downshift, some of which will be of relatively high magnitude. In this case, any prominent synapomorphy of that lineage may appear to be linked to a diversification shift by sporadic association.

**Fig. 2:**
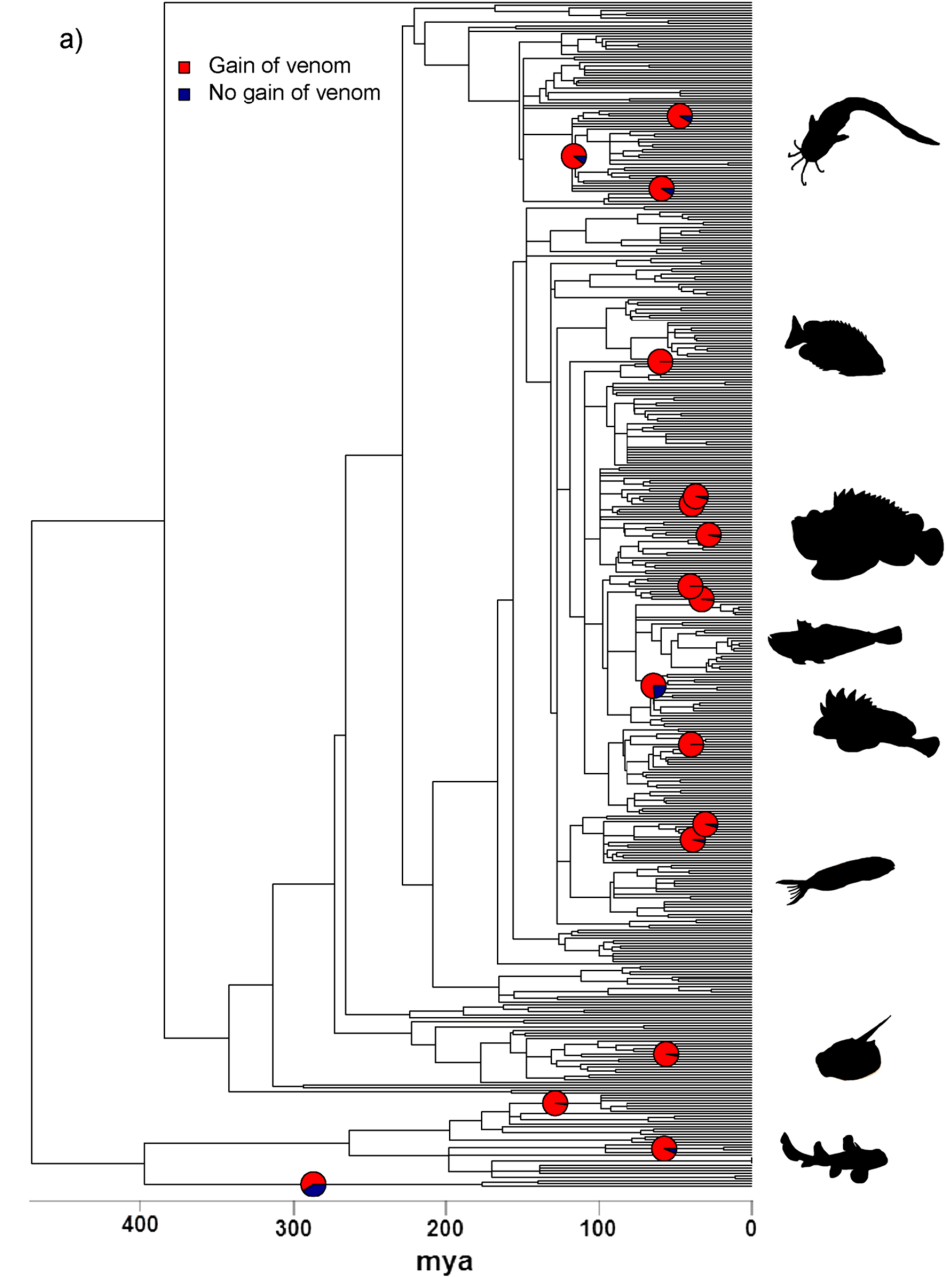

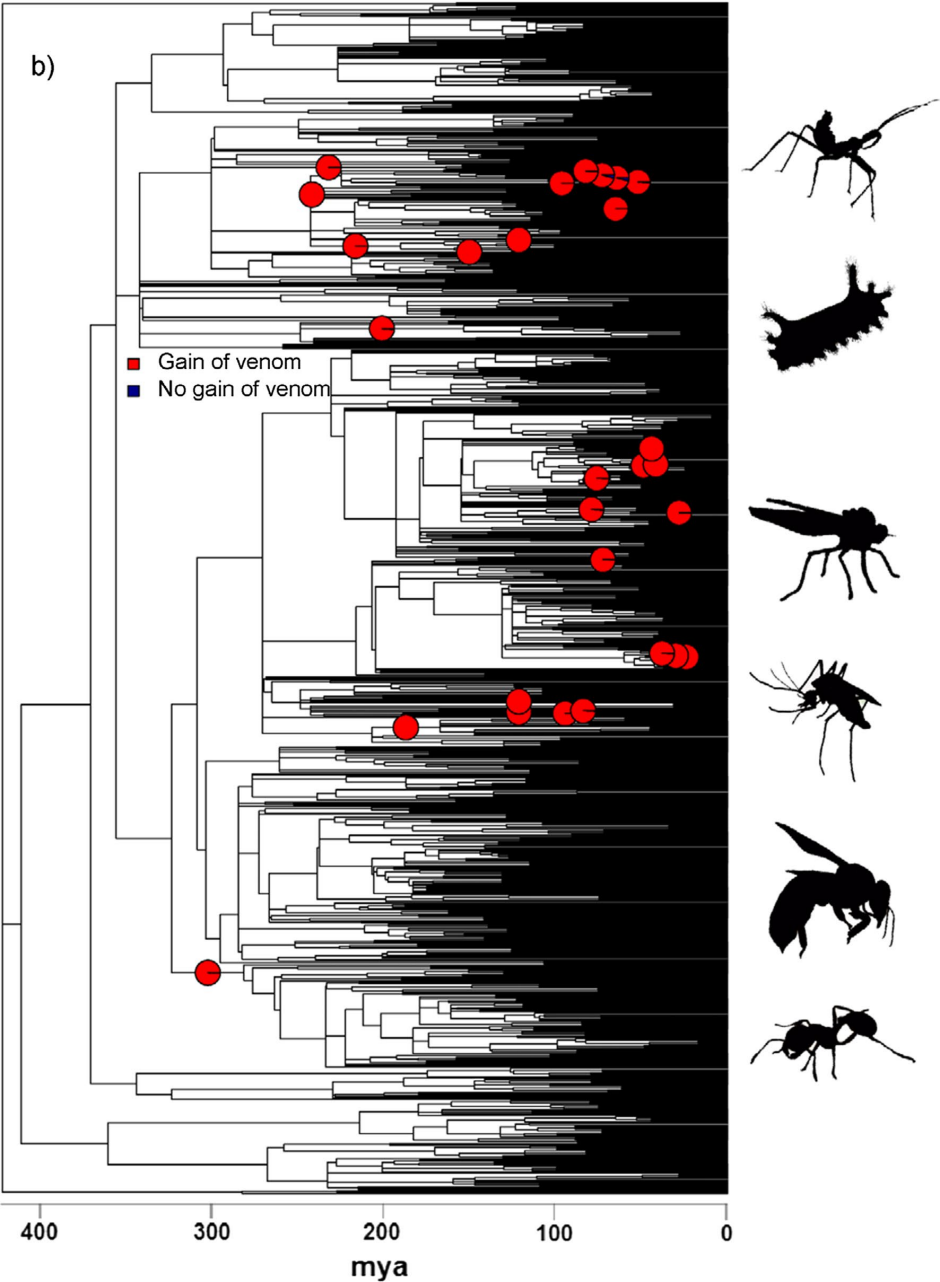
Gains of venom in fishes (**a**) and insects (**b**) based on ancestral state estimation. Pie charts represent the probability of a gain of venom where there is a minimum of P = 0.2 that the gain happened on that branch. Although the scale of the phylogenies prevents showing the tip labels we have included separate pdf versions of these plots as Additional file 2: Fig. S3, Additional file 3: Fig. S4, which contain visible Family names when enlarged. Representative fish species depicted at the right of the phylogeny are, from top to bottom, *Plotosus lineatus* (Plotosidae), Siganus fuscescens (Siganidae), *Synanceia horrida* (Synanceiidae), *Echiichthys vipera* (Trachinidae), *Paracentropogon rubripinnis* (Tetrarogidae), *Meiacanthus grammistes* (Blenniidae), *Neotrygon kuhlii* (Dasyatidae), and *Heterodontus francisci* (Heterodontidae). Representative insect species shown are, from top to bottom, *Pristhesancus plagipennis* (Reduviidae), *Acharia stimulea* (Limacodidae), *Dysmachus trigonus* (Asilidae), *Aedes albopictus* (Culicidae), *Vespula vulgaris* (Vespidae), and *Solenopsis invicta* (Formicidae)

Ancestral state reconstructions showed frequent origins of venom in both fishes and insects (Fig. 2). Specifically, we estimated a minimum of 19–20 origins of venom in fishes (17 of which are strongly supported on a particular branch and are plotted in Fig. 2), which is consistent with previous estimates for fishes [24, 26], and a minimum of 28 origins in insects. In contrast to the situation in tetrapods [14] we find that gains of venom are more frequent than losses in both fishes (19–20 gains cf. 11 losses) and insects (28 gains cf. 2 losses). This may be related to a more prominent role in defence in both groups compared to most venomous tetrapods since chemically defended amphibians are also more likely to gain than lose their poison [17]. However, additional research investigating which factors drive the gains and losses of venom in different taxonomic groups is required to provide a strong explanation for these patterns. Finally, our ancestral state estimates reveal that a substantial proportion of the evolutionary history of both fishes (~ 10%) and insects (~ 14%) has been spent in a venomous state, providing ample opportunity for effects on diversification rates to contribute markedly to the extant species richness of both groups.

The temporal pattern of venom origins appears to differ between the two groups considered here: in insects gains of venom are spread across the history of the clade, but in fishes the majority of origins are concentrated into two narrow bands (Fig. 2). These time periods correspond to the Late Cretaceous and the Eocene. Intriguingly, an Eocene burst of independent gains in venom would correspond to the origin and a diversity peak of cetaceans [63, 64], whilst Late Cretaceous origins coincide with the origin and diversification of mosasaurs and a decline of other large Mesozoic marine predators [65, 66]. Moreover, like cetaceans, mosasaurs are thought to have been active and endothermic predators [67], and locomotor styles between these two predatory groups are also thought to be similar [68]. Although we do so cautiously due to the limitations of our family-level resolution and lack of direct evidence, we speculate that most origins of fish venom were linked to similar predation pressures by mosasaurs in the Late Cretaceous and cetaceans in the Eocene.

Finally, we note that recent studies of diversification patterns have uncovered a biogeographic signal of time-for-speciation due to different times of colonisation of different geographic areas, as well as diversity gradients [69, 70]. Given the many origins of venom in both fishes and insects, and the very wide distributions of many families (global or hemisphere-wide in many cases) which are venomous, there is no clear indication that geographic patterns would confound our results here. However, because of the family-level scale of our analyses and the wide distribution of many families, robustly estimating the biogeographical origin for each of those families to obtain a strong basis for proper testing of geographical effects would likely require species-level analyses and is beyond the scope of the current study. Nevertheless, more generally the biogeography of venom evolution, particularly in the context of distributions of natural enemies [71, 72], and interactions with diversification is likely to be a fruitful area for future studies.

We provide the first broad-scale family-level test of whether venom has contributed to the enormous diversity contained within the superradiations of insects and fishes. We first showed that diversification rate variation is an important determinant of species richness in both of these groups, and then demonstrated that venom is associated with higher diversification rates. We highlight that venom has evolved many times in both insects and fishes, and that a substantial proportion of their evolutionary history has been spent in possession of venom. Consequently, our results provide evidence that venom has played a part in generating the diversity in the largest vertebrate and invertebrate animal radiations.

## Supplementary Information

**Additional file 1**. Additional figures and tables.

**Additional file 2: Figure S3**. Estimate of gains of fish venom - tip labels.

**Additional file 3: Figure S4**. Estimate of gains of insect venom - tip labels.

## Data Availability

The datasets generated and analysed during the current study, as well as the R code used, are available in the FigShare repository, https://figshare.com/s/37be6229b8d29fd7e06c.

## Abbreviations

AIC: Akaike’s information criterion.
ARD: All rates different.
ΔAIC: Difference between AIC of a given model and the best model in the dataset.
OU: Ornstein–Uhlenbeck
