# Supplementary material for: Radiating pain: venom has contributed to the diversification of the largest radiations of vertebrate and invertebrate animals
Source: BMC Ecol Evol. 2021 Aug 3;21:150. doi: 10.1186/s12862-021-01880-z (PMC8336261; doi:10.1186/s12862-021-01880-z)
Supplement: Supplementary file 1 — Additional file 1. Additional figures and tables. [file 12862_2021_1880_MOESM1_ESM.docx]

**Supplementary Material for ‘Radiating Pain: Venom has Contributed to the Diversification of the Largest Radiations of Vertebrate and Invertebrate Animals’**

| *Fishes* | Coefficient | SE | t | P |
| --- | --- | --- | --- | --- |
| Intercept | -1.2518 | 0.8616 | -1.4529 | 0.1470 |
| Venom | 0.5321 | 0.2321 | 2.2923 | 0.0224 |
| Diversification rate (ϵ=0) | 24.8800 | 1.2192 | 20.4063 | <2.2x10^-16^ |
| Clade age | 0.0464 | 0.0051 | 9.1847 | <2.2x10^-16^ |
| Clade age (quadratic) | -0.0001 | 0.00002 | -7.0028 | 9.1x10^-12^ |
|  |  |  |  |  |
| Intercept | 0.3065 | 0.6334 | -0.4840 | 0.6286 |
| Venom | 0.4874 | 0.2106 | 2.3141 | 0.0211 |
| Diversification rate (ϵ=0.9) | 47.1520 | 2.0887 | 22.5750 | <2.2x10^-16^ |
| Clade age | 0.0341 | 0.0041 | 8.2356 | 1.9x10^-15^ |
| Clade age (quadratic) | -0.0001 | 0.00002 | -6.4400 | 3.0x10^-10^ |
|  |  |  |  |  |
| *Insects* | Coefficient | SE | t | P |
| Intercept | -1.4047 | 0.4233 | -3.3184 | 0.0009 |
| Venom | 0.6082 | 0.1774 | 3.4293 | 0.0006 |
| Diversification rate (ϵ=0) | 35.5730 | 1.6189 | 21.9745 | <2.2x10^-16^ |
| Clade age | 0.0530 | 0.0048 | 10.9829 | <2.2x10^-16^ |
| Clade age (quadratic) | -0.0001 | 0.00001 | -9.0003 | <2.2x10^-16^ |
|  |  |  |  |  |
| Intercept | 0.1223 | 0.3760 | 0.3251 | 0.7451 |
| Venom | 0.7334 | 0.1713 | 4.2813 | 0.00002 |
| Diversification rate (ϵ=0.9) | 45.5580 | 2.0723 | 21.9843 | <2.2x10^-16^ |
| Clade age | 0.0399 | 0.0045 | 8.8385 | <2.2x10^-16^ |
| Clade age (quadratic) | -0.0001 | 0.00001 | -7.1411 | 1.9x10^-12^ |

**Table S1 –** Model output predicting species richness for diversification rate based on ϵ=0 and ϵ=0.9. Lambda parameter for fish models was estimated to be 0.814 (ϵ=0) and 0.634 (ϵ=0.9). The estimated parameters from the OU model for insects were α=0.014 (phylogenetic half-life of 50.3my) and σ^2^=0.088 (ϵ=0), and α=0.016 (phylogenetic half-life of 42.5my) and σ^2^=0.104 (ϵ=0.9).


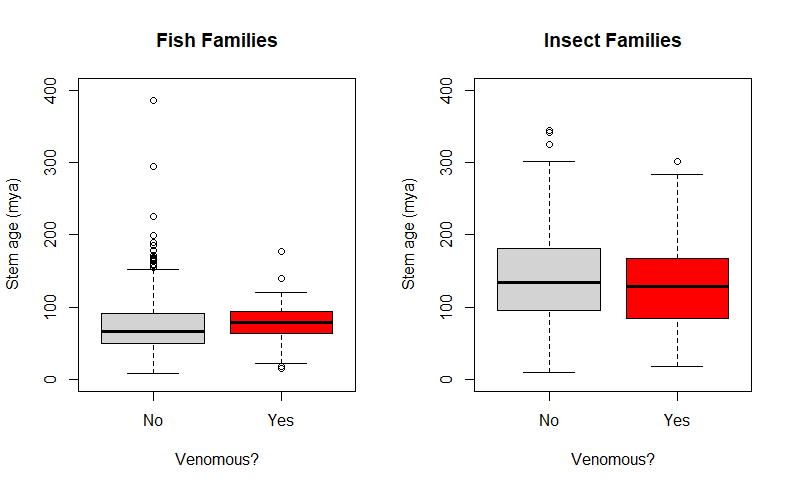


**Figure S1 –** Stem ages of venomous vs non-venomous families for fishes and insects.


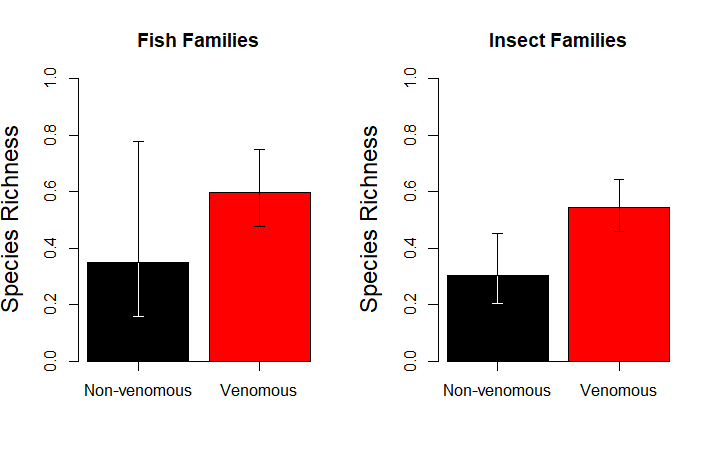


**Figure S2 –** Independent effect of venom on species richness of fish and insect families after accounting for diversification rate and clade age (based on estimated parameters from the model including standard error). Note that although statistically significant the additional effect is very small because most of the variation is explained by diversification rate and age.

| *Fishes* | Coefficient | SE | t | P |
| --- | --- | --- | --- | --- |
| Intercept (ϵ=0) | 0.1212 | 0.0067 | 18.1840 | <2.2x10^-16^ |
| Venom | 0.0153 | 0.0067 | 2.2662 | 0.0239 |
| Clade age | -0.0012 | 0.0001 | -10.4023 | <2.2x10^-16^ |
| Clade age (quadratic) | 3.0x10^-6^ | 4.5x10^^-7^ | 6.6571 | 8.1x10^-11^ |
|  |  |  |  |  |
| Intercept (ϵ=0.9) | 0.0512 | 0.0037 | 13.9536 | <2.2x10^-16^ |
| Venom | 0.0087 | 0.0038 | 2.2504 | 0.0249 |
| Clade age | -0.0005 | 0.0001 | -7.7494 | 6.1x10^-14^ |
| Clade age (quadratic) | 1.2x10^-6^ | 2.5x10^-7^ | 4.8102 | 2.1x10^-6^ |
|  |  |  |  |  |
| *Insects* | Coefficient | SE | t | P |
| Intercept (ϵ=0) | 0.1559 | 0.0230 | 6.7900 | 2.1x10^-11^ |
| Venom | 0.0212 | 0.0062 | 3.4236 | 0.0006 |
| Clade age | -0.0013 | 0.0001 | -11.543 | <2.2x10^-16^ |
| Clade age (quadratic) | 3.0x10^-6^ | 3.4x10^-7^ | 8.9211 | <2.2x10^-16^ |
|  |  |  |  |  |
| Intercept (ϵ=0.9) | 0.0887 | 0.0182 | 4.8709 | 1.3x10^^-6^ |
| Venom | 0.0070 | 0.0049 | 1.4236 | 0.1549 |
| Clade age | -0.0008 | 0.0001 | -8.3932 | <2.2x10^-16^ |
| Clade age (quadratic) | 1.7x10^-6^ | 2.7x10^-7^ | 6.4717 | 1.6x10^-10^ |

**Table S2 –** Model output predicting diversification rate based on ϵ=0 and ϵ=0.9. The estimated parameters from the OU model for fish models were α=0.046 (phylogenetic half-life of 15.1my) and σ^2^=0.0002 (ϵ=0), and α=0.070 (phylogenetic half-life of 9.9my) and σ^2^=8.5x10^-5^ (ϵ=0.9). Kappa was estimated to be 3.4x10^-8^ (ϵ=0) and 3.6x10^-8^ (ϵ=0.9) for insects. Note that venom is not a significant predictor of diversification rate in insects if we assume very high relative extinction rates (0.9), however we expect relative extinction rate to be much lower than this based on current understanding (see main text for more detail).

| *Fishes* | Coefficient | SE | t | P |
| --- | --- | --- | --- | --- |
| Intercept | 0.0965 | 0.0058 | 16.5180 | <2.2x10^-16^ |
| Venom | 0.0104 | 0.0069 | 1.5095 | 0.0132 |
| Clade age | -0.0010 | 0.0001 | -9.3286 | <2.2x10^-16^ |
| Clade age (quadratic) | 2.3x10^-6^ | 3.9x10^-7^ | 5.9193 | 6.5x10^-9^ |
|  |  |  |  |  |
| *Insects* | Coefficient | SE | t | P |
| Intercept | 0.1355 | 0.0216 | 6.2739 | 5.5x10^-10^ |
| Venom | 0.0205 | 0.0059 | 3.4671 | 0.0006 |
| Clade age | -0.0012 | 0.0001 | -10.7353 | <2.2x10^-16^ |
| Clade age (quadratic) | 2.7x10^-6^ | 3.2x10^-7^ | 8.3055 | 3.7x10^-16^ |

**Table S3 –** Model output predicting diversification rate based on ϵ=0.5 on a reduced dataset excluding all families with interspecific variation in whether they are venomous or not. The estimated parameters from the OU model for fish models were α=0.050 (phylogenetic half-life of 13.9my) and σ^2^=0.0001, and kappa was estimated to be 3.5x10^-8^ for insects. These results are consistent with the same reduced dataset when analysed using the richness Yule test, which again found higher diversification rates in venomous families of both fishes (*χ*^2^=15.51, df=1, P=8.2x10^-5^) and insects (*χ*^2^=12.34, df=1, P=0.0004).

| Model set | Model | AIC | ΔAIC |
| --- | --- | --- | --- |
| Fish species richness (ϵ=0.5) | Lambda | 1449.81 | 0.00 |
|  | OUrandom | 1469.95 | 20.14 |
|  | OUfixed | 1469.95 | 20.14 |
|  | Kappa | 1505.25 | 55.44 |
|  | GLM | 1511.32 | 61.51 |
|  | Delta | 1569.91 | 120.10 |
|  | Trend | 1607.05 | 157.24 |
|  | BM | 1607.11 | 157.30 |
|  | EB | 1609.11 | 159.30 |
| Insect species richness (ϵ=0.5) | OUrandom | 3516.23 | 0.00 |
|  | OUfixed | 3516.23 | 0.00 |
|  | Lambda | 3563.26 | 47.02 |
|  | Delta | 3569.36 | 53.13 |
|  | Kappa | 3593.35 | 77.11 |
|  | GLM | 3594.40 | 78.17 |
|  | BM | 3651.41 | 135.18 |
|  | Trend | 3652.41 | 136.18 |
|  | EB | 3653.41 | 137.18 |
| Fish diversification rates (ϵ=0.5) | OUrandom | -1717.11 | 0.00 |
|  | OUfixed | -1717.11 | 0.00 |
|  | Lambda | -1700.04 | 17.07 |
|  | Kappa | -1681.26 | 35.85 |
|  | GLM | -1679.61 | 37.50 |
|  | Delta | -1382.44 | 334.67 |
|  | BM | -1298.80 | 418.31 |
|  | Trend | -1297.68 | 419.43 |
|  | EB | -1296.80 | 420.31 |
| Insect diversification rates (ϵ=0.5) | Kappa | -3469.95 | 0.00 |
|  | OUrandom | -3450.41 | 19.55 |
|  | OUfixed | -3450.41 | 19.55 |
|  | Lambda | -3308.49 | 161.47 |
|  | GLM | -3247.18 | 222.78 |
|  | Delta | -3091.54 | 378.41 |
|  | BM | -2810.39 | 659.56 |
|  | Trend | -2808.99 | 660.96 |
|  | EB | -2808.39 | 661.56 |
| BiSSE (fishes) | μ constrained | 4875.84 | 0.00 |
|  | HiSSE model | 4912.94 | 37.10 |
|  | μ+λ constrained | 5248.87 | 373.03 |
|  | full model | 5250.13 | 374.29 |
|  | λ constrained | 5250.91 | 375.07 |
|  | all constrained | 5254.13 | 378.29 |
| BiSSE (insects) | μ constrained | 10658.77 | 0.00 |
|  | HiSSE model | 10659.19 | 0.42 |
|  | μ+λ constrained | 11084.92 | 426.15 |
|  | all constrained | 11085.99 | 427.22 |
|  | λ constrained | 11087.57 | 428.80 |
|  | full model | 11087.86 | 429.09 |

**Table S4 –** (Δ)AIC values for each model set; phylogenetic logistic regressions based on ϵ=0.5 but other values were almost identical in terms of ΔAIC values and so are not shown here for brevity/clarity. Within each model set the models are ordered by descending AIC (better supported model at the top).


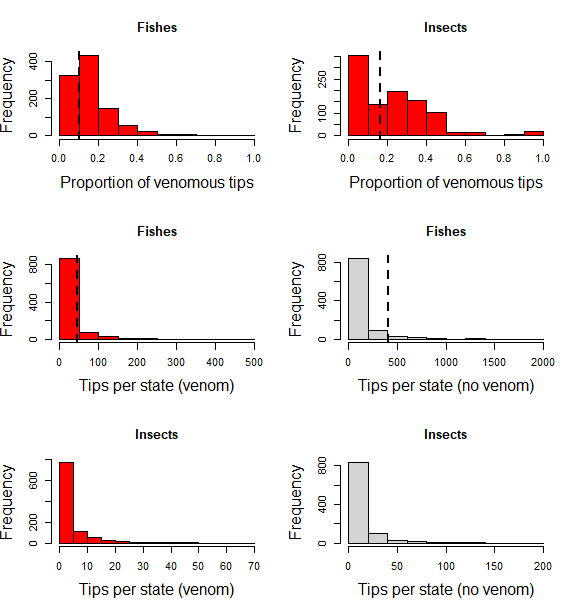


**Figure S5 –** Model adequacy checks of the best BiSSE models for each taxon (in both cases this was a model of equal extinction rates but different speciation rates between states). Histograms represent 1000 simulations under the best model and vertical dashed lines represent the observed values in the dataset. Although the models generate the observed proportions of venomous species, and are not too divergent in tips per state for fishes, they greatly underestimate the total number of species of insects (dashed lines in the bottom row are well outside the bounds of the plot).
