# Supplementary figures and images for: Radiating pain: venom has contributed to the diversification of the largest radiations of vertebrate and invertebrate animals
Source: BMC Ecol Evol. 2021 Aug 3;21:150. doi: 10.1186/s12862-021-01880-z (PMC8336261; doi:10.1186/s12862-021-01880-z)

- Gain of venom
- No gain of venom

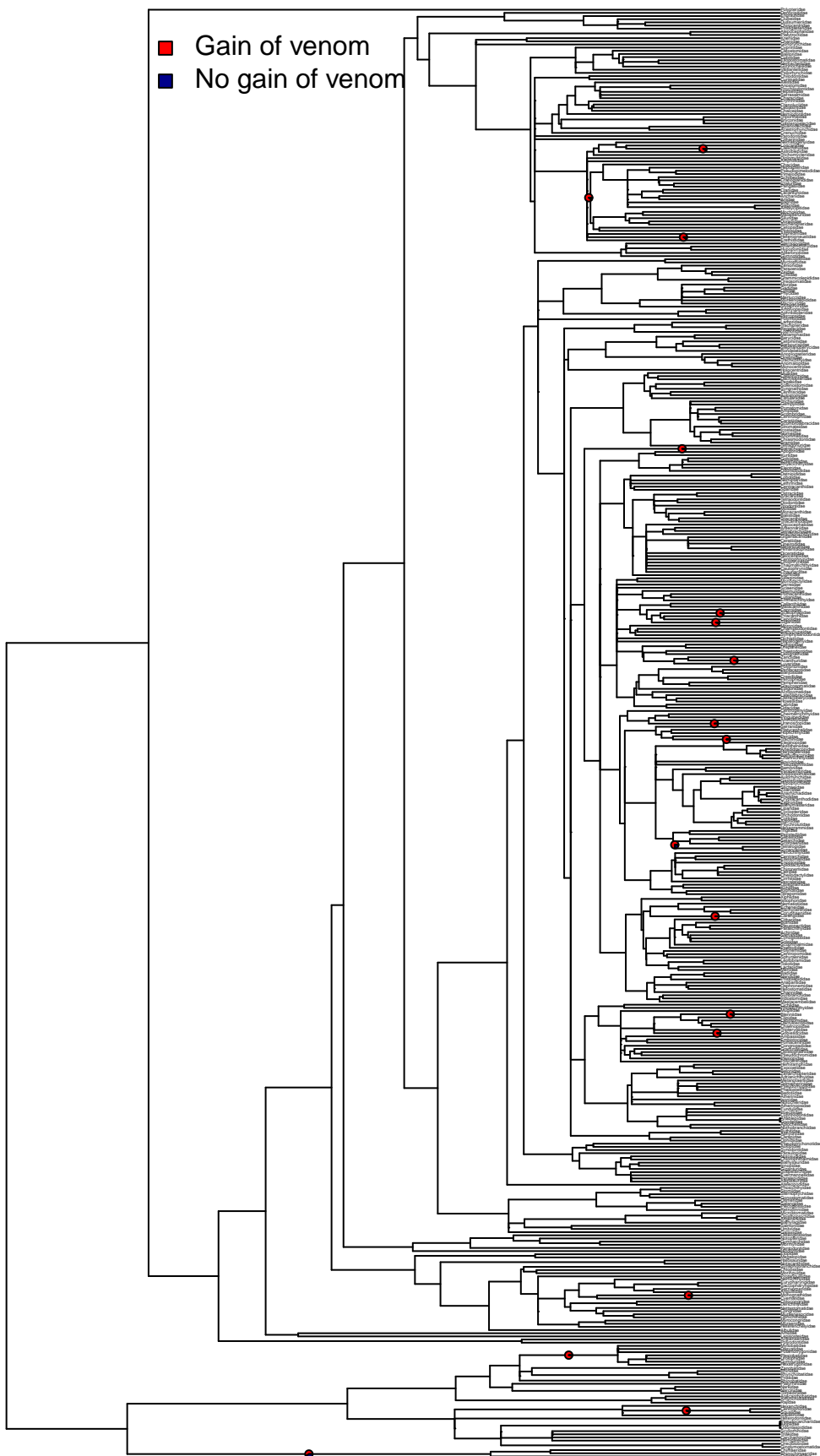

Supplement: Supplementary file 2 — Additional file 2: Figure S3. Estimate of gains of fish venom - tip labels. [file 12862_2021_1880_MOESM2_ESM.pdf]
