# Supplementary material for: Radiating pain: venom has contributed to the diversification of the largest radiations of vertebrate and invertebrate animals
Source: BMC Ecol Evol. 2021 Aug 3;21:150. doi: 10.1186/s12862-021-01880-z (PMC8336261; doi:10.1186/s12862-021-01880-z)
Supplement: Supplementary file 3 — Additional file 3: Figure S4. Estimate of gains of insect venom - tip labels. [file 12862_2021_1880_MOESM3_ESM.pdf]

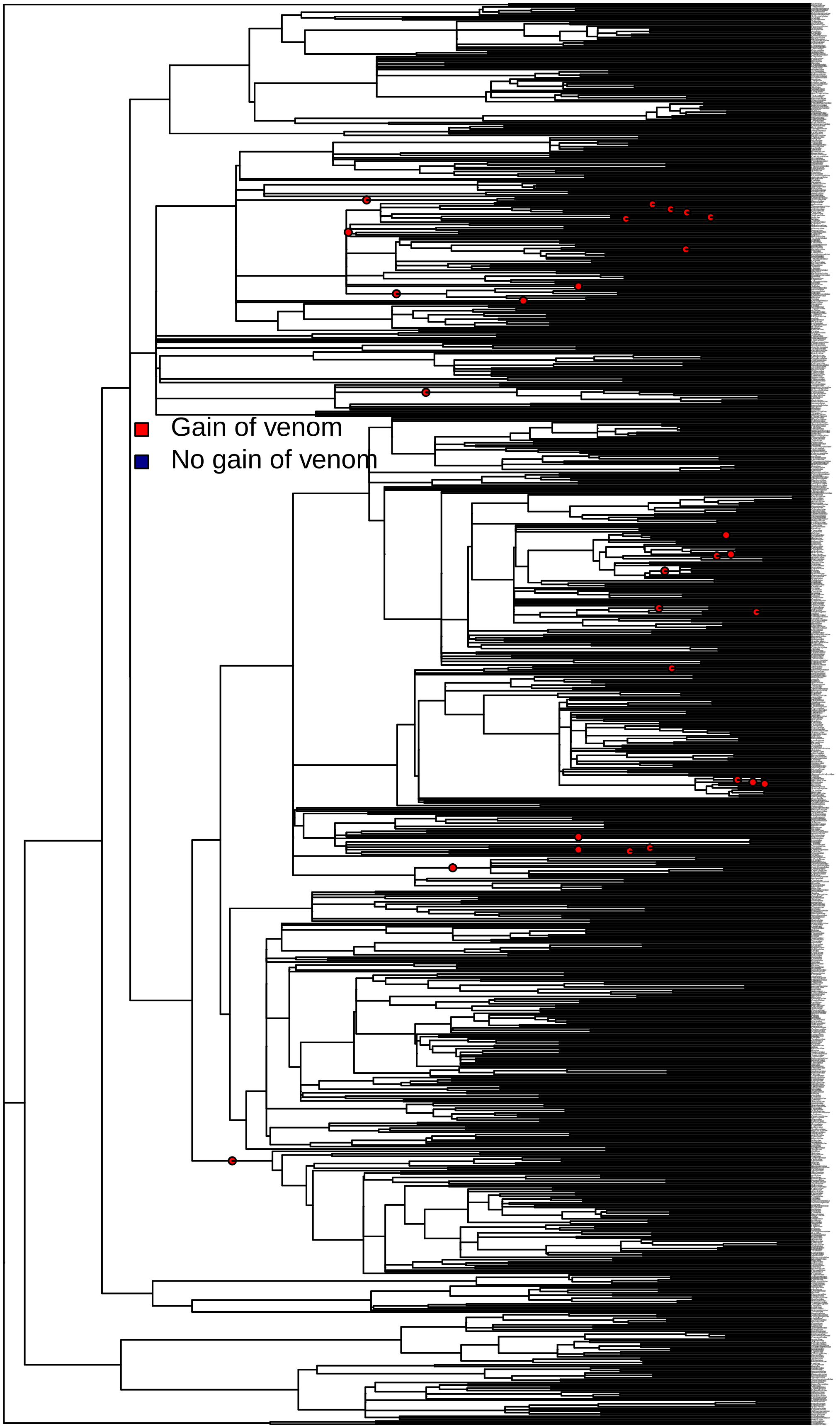
Gain of venom
No gain of venom
